# The Potential of Two Phytoseiid Mites as Predators of the Grape Erineum Mite, *Colomerus vitis*

**DOI:** 10.3390/plants13141953

**Published:** 2024-07-17

**Authors:** Mahmoud M. Al-Azzazy, Saleh S. Alhewairini

**Affiliations:** 1 Department of Plant Protection, College of Agriculture and Food, Qassim University, P.O. Box 6622, Buraidah 51452, Saudi Arabia; m.elazzazy@qu.edu.sa; 2 Department of Agricultural Zoology and Nematology, College of Agriculture, Al-Azhar University, Cairo 11651, Egypt

**Keywords:** biological control, *Phytoseius plumifer*, *Euseius scutalis*, mixed diet, alternative food

## Abstract

*Phytoseius plumifer* (Canestrini and Fanzago) and *Euseius scutalis* (Athias-Henriot) (Phytoseiidae) are generalist predatory mites important in controlling phytophagous mites on some agricultural crops. The biology of both species as potential biological control agents of the grape erineum mite, *Colomerus vitis* (Pagenstecher) (Eriophyidae) on grape leaf disks was studied in the laboratory at 33 ± 1 °C, 60%RH, 12:12 h L:D. The developmental time, survival, and reproductive parameters of *P. plumifer* and *E. scutalis* on *C. vitis*, date palm pollen as well as *C. vitis* plus date palm pollen were investigated. Both predators, *P. plumifer* and *E. scutalis*, thrived on the mixed diet of *C. vitis* and date palm pollen resulting in a shorter developmental time (6.16 and 6.69 days, respectively), higher oviposition rate (2.11 and 1.96 eggs/female/day, respectively), and higher intrinsic rate of increase (0.251 and 0.229 per female/day, respectively) than on any other diet. Date palm pollen was an adequate alternative food source for *P. plumifer* and *E. scutalis*. The results suggest that both predators have good potential to suppress *C. vitis* populations and that date palm pollen can support the population establishment of both predators in the absence or scarcity of the main prey in the environment. We discuss the relevance of our results for the biocontrol of *C. vitis*.

## 1. Introduction

Grapevine (*Vitis vinifera* L.) is an economically important fruit crop in the world and, globally, the third most valuable horticultural crop after potatoes and tomatoes [1]. Worldwide vineyard surface accounts for 7.327 million hectares, with a total grape production of 77.8 Mio.t that sustains dried grape and fresh grape markets and wine elaboration worldwide. However, most grapevine cultivars are susceptible to mite infestations, which considerably limit grape production. Moreover, grape mite infestations are becoming worse owing to the excessive use of pesticides and the destruction of biodiversity in recent years [2]. It is well known that some serious mite species occur on grape leaves. One of the most devastating pests globally infecting grapevines is grape erineum mite, *Colomerus vitis* (Pagenstecher), which may result in total crop damage in case of severe infestation [3]. The feeding of this mite causes a decrease in leaf area, photosynthetic rate, and chlorophyll contents and an increase in leaf fresh weight due to the hyperplasia and hypertrophy of mesophyll and epidermal cells and leaf deformities as well as reduction in the growth of green grapevine shoots and causes damage in nurseries and vineyards [4,5,6,7]. *Colomerus vitis*-infested grape leaves initially show white patches on the lower leaf surface. The young leaves then twist, become fragile, and experience the formation of a layer of white fluff plaque at the back, which appears blister-like on the upper surface. Analogous to rusts, yellow, fungi-like spots form in the subsequent period and, finally, become reddish brown. When *C. vitis* infestation is severe, the grapevines cannot renew and grow new buds, which inhibits the ability of leaves, and it affects grape production [8]. Furthermore, *C. vitis* infestation in grape leaves causes the spread of grapevine pinot gris virus (GPGV) [5,9], as well as grape berry necrosis virus (GINV) [9]. Therefore, grape plant health management is needed which enhances productivity, prevents crop loss, and contributes towards food security.

Currently, pest management practices are generally limited to chemical pesticide application; vineyards are subjected to very high levels of synthetic pesticides [10]. The use of synthetic pesticides to manage phytophagous mites results in the resistance of mites to major pesticides, the resurgence of secondary diseases and pests, the lethal effects of pesticides on natural enemies and other non-target organisms, and the presence of pesticide residues in crops, as well as negative impacts on biodiversity and on the environment and human health [11,12,13]. These negative impacts strongly limit the sustainability of farming systems and primarily necessitate the development of non-chemical methods of pest control. Additionally, due to concerns regarding the adverse effects of chemical pesticides, there is an increasing demand for pesticide-free fruits. Consequently, it is imperative to find effective biological control agents against *C. vitis* [3]. The utilization of predatory mites as a tool in phytophagous mites’ management is important for sustainability and food security and a promising way to reduce the level of chemical pesticide use [14,15]. Phytoseiid mites are the most extensively studied and most applied biocontrol agents of phytophagous mites (particularly of the Eriophyoidea and Tetranychoidea), thrips and whiteflies in orchards, grapes, citrus crops, and greenhouses [16,17,18,19,20]. Most of all, phytoseiids are generalist predators and can survive and develop feeding on prey when they are available but are also capable of surviving on a broad range of other foods (plant exudates, pollen, nectar, fungi, etc.) when the prey are rare or absent [21]. *Phytoseius plumifer* (Canestrini and Fanzago) (Phytoseiidae) is an important generalist predator, and one of the most abundant natural enemies and an efficient predator of phytophagous mites on various crops in several countries [22,23]. It is found naturally on grapes in many countries around the world [19]. This predator is capable of preying on a range of prey-food types, including eriophyid mites, tetranychids, tarsonemids, and pollen as food [15,24,25,26]. Also, *Euseius scutalis* (Athias-Henriot) (Phytoseiidae) is a predator that can be considered in integrated pest management (IPM) programs against some pests. It appears to be adapted to several plants, such as fig, pomegranate, apple, okra, vines, avocado, citrus, etc. [27,28]. *E. scutalis* is a generalist predator capable of preying on a wide range of food items, including eriophyid mites, tetranychid mites, whiteflies, scale insects, and the eggs of some insects, in addition to pollen grains [29,30,31,32,33].

Habitually, generalist predacious mites can exploit different diets, including natural prey and pollen [34,35]. Furthermore, plant pollens can serve as a supply of nutrients and water complementary to a diet consisting of prey [36,37]. Many studies have shown that adding pollen to a crop can promote pest control by predacious mites [38]. The pollinivory of *P. plumifer* and *E. scutalis* offers the possibility to pre-establish populations in vineyards with the supplementation of pollen and allows them to sustain their populations in the grape crops when prey is scarce or absent and thus prevent severe declines in predatory mite populations during scarcities of primary prey. So far, no study has examined the potential of *P. plumifer* and *E. scutalis* as predators of the grape erineum mite, *C. vitis*. The main objective of the current laboratory study was to compare the potential of these predatory mite species as biocontrol agents of *C. vitis*. In particular, the developmental, survival, predation rate, and reproductive performance of the two phytoseiid mites was assessed, using *C. vitis*, date palm pollen, or a combination of both as food.

## 2. Results

### 2.1. Developmental Time and Survival of Immature Stages

*Phytoseius plumifer* and *Euseius scutalis* developed successfully on *C. vitis*, *date palm pollen,* and *C. vitis* plus date palm pollen. The mites developed the fastest when reared on *C. vitis* plus date palm pollen and the slowest on date palm pollen only (Table 1). That diet significantly affected the developmental time of *E. scutalis* but not that of *P. plumifer*. The predatory mite, *P. plumifer,* completed its development faster as compared to *E. scutalis* on all three diets. *P. plumifer* showed similar egg-to-adult developmental times of 6.16–6.37 days on the three diets, while *E. scutalis* required 6.69–8.44 days. Diet had no significant effect on the durations of the different developmental stages of *P. plumifer*. On the contrary, the predatory mite, *E. scutalis*, developed significantly faster on a mixed diet as compared to date palm pollen alone (*p* < 0.005) but not as compared to *C. vitis* alone (*p* = 0.130). The stages of this species developed faster on *C. vitis* alone as compared to date palm pollen alone (*p* = 0.021) but not as compared to a mixed diet (*p* = 0.845) (Table 1). On all tested diets, the survival of the immature stages was as high as 94.12 for *P. plumifer* and 91.80 for *E. scutalis* (Table 2). The interaction between diet and predator species was not significant, and there was not a noteworthy effect of predator species on survival from egg to adult. Tukey’s HSD test indicated that juvenile survival was not significantly affected by diet.

### 2.2. Adult Longevity

Overall, no significant difference in the pre-oviposition period was observed between *C. vitis* and pollen alone, whereas the shortest pre-oviposition period was recorded on a mixed diet for *P. plumifer* and *E. scutalis*. (Table 3). Diet had a clear significant effect on the generation period and female longevity in both predator species. There were insignificant differences between the pollen and *C. vitis* and *C. vitis* diet treatments (*p* = 0.0274), while there was a significant difference between treatments of pollen diet alone, *C. vitis* and pollen, and *C. vitis* (*p* = 0.0243). The generation period and female adult longevity lasted 9.22 and 28.48 days, 9.08 and 33.13 days, and 8.37 and 33.43 days when *P. plumifer* fed on date palm pollen, *C. vitis*, and *C. vitis* plus pollen, respectively. The corresponding periods were 12.81 and 24.11 days, 10.40 and 28.30 days, and 9.14 and 28.96 days when *E. scutalis* fed on date palm pollen, *C. vitis*, and *C. vitis* plus pollen, respectively. For both predators, the oviposition period differed significantly among food types (*p* = 0.064). When the mixed diet was the food source, the total oviposition period was the longest, whereas it was the shortest when the predators fed on pollen alone (Table 3).

### 2.3. Reproduction

*Phytoseius plumifer* and *E. scutalis* showed similar fecundity on either diet (*p* = 0.8543). In contrast, in both predatory mites, fecundity was significantly affected by diet (*p* < 0.001). On the grape erineum mite diet, total oviposition over the oviposition period averaged 53.94 ± 1.28 and 40.09 ± 1.22 eggs for *P. plumifer* and *E. scutalis*, respectively (*p >* 0.878). A combination of *C. vitis* and date palm pollen resulted in an average oviposition of 56.81 ± 1.30 and 44.16 ± 1.35 eggs over the oviposition period for the respective predators (*p >* 0.985). The addition of date palm pollen to the diet of *C. vitis* substantially increased the reproductive performance of both *P. plumifer* (*p* = 0.004) and *E. scutalis* (*p* < 0.001). In addition, *P. plumifer* and *E. scutalis* females fed on pollen alone exhibited a lower rate of fecundity than those feeding on grape erineum mite diet and the mixed diet. The post-oviposition periods of *P. plumifer* and *E. scutalis* did not differ between the three tested diets (Table 4).

### 2.4. Predation of P. plumifer and E. scutalis

The larvae of both predators were inactive and did not feed during the experiment, and the feeding activity started immediately after the predators entered the protonymphal stages. Both *P. plumifer* and *E. scutalis* successfully suppressed the population of *C. vitis* on the small laboratory-rearing units, in the absence of date palm pollen. The mean daily predation rate was significantly affected by predator age and diet (*p* < 0.001).

For both predators, the adults consumed more prey compared with the nymph stages. There was a significant difference in the predation rate of adult *C. vitis* between the two species of predatory mites, *P. plumifer* and *E. scutalis*. The total number of *C. vitis* prey consumed by *P. plumifer* and *E. scutalis* immature and adult stages are shown in (Table 5 and Table 6). In the absence of date palm pollen, immature females of *P. plumifer* significantly consumed a higher number of prey 106.86 ± 3.64 than *E. scutalis* 96.55 ± 2.17. The highest means for the daily predation of females were observed during the oviposition period, with the female of *P. plumifer* devouring an average of 2931.40 ± 16.62, while the female of *E. scutalis* consumed an average of 1994.52 ± 12.40. Thereafter, the daily consumption of predators fed on *C. vitis* decreased with age. At the end of the experiment, *P. plumifer* showed the highest predation rates on *C. vitis* in the absence of date palm pollen. The highest number of preys consumed during the life span was reported for *P. plumifer* females with 3525.63 ± 16.47 prey, while for *E. scutalis,* it was 2452.49 ± 10.53 prey (Table 5). So, it could be concluded that *P. plumifer* performance was better than *E. scutalis* against *C. vitis*. Providing date palm pollen with *C. vitis* resulted in a significant reduction in total prey consumption during *P. plumifer* and *E. scutalis* juvenile development from approx. 106.86 ± 3.64 and 96.55 ± 2.17 mites per predator female when mites were reared on *C. vitis* only to approx. 56.74 ± 1.47 and 46.60 ± 1.38 mites per predator female in the mixed diets for *P. plumifer* and *E. scutalis*, respectively. A similar trend was found for both predators during female longevity periods. Providing date palm pollen with *C. vitis* resulted in a significant reduction in the total prey consumption during *P. plumifer* and *E. scutalis* longevity from 3418.77 ± 14.50 and 2355.87 ± 15.85 mites per predator when mites were reared on *C. vitis* only to approx. 2052.00 ± 11.34 and 1247.65 ± 16.25 mites per predator in the mixed diets for *P. plumifer* and *E. scutalis*, respectively. Similarly, the highest means for the daily consumption rate of females were observed throughout the oviposition period, with the female of *P. plumifer* devouring an average of 1769.43 ± 10.42, while the female of *E. scutalis* devoured an average of 1037.83 ± 3.52. The highest number of preys consumed during the life span reported for *P. plumifer* females was 2108.74 ± 14.23 prey, while for *E. scutalis,* it was 36.56 ± 2.30 prey (Table 6).

### 2.5. Population Growth Parameters

Based on the above-mentioned findings, we calculated the life table parameters of *P. plumifer* and *E. scutalis* for each treatment. The highest life table parameter value was recorded when the predators were fed the mixed diet of *C. vitis* and date palm pollen. For both predators, the females reared on the mixed diet of *C. vitis* and date palm pollen showed the highest net reproductive rate (*R_o_*), intrinsic rate of natural increase (*r_m_*), and finite rate of increase (λ). On the other hand, *P. plumifer* and *E. scutalis* individuals reared on the mixed diet had the shortest mean generation time (Table 7).

### 2.6. Sex Ratio

The diet had a clear significant effect on the sex ratio in both predators. As shown in Table 7, sex ratio on all diets ranged from 53 to 76%. There were insignificant differences between the treatments of mixed diet (pollen plus *C. vitis*) and *C. vitis* diet alone. Also, there was a significant difference between treatments of pollen diet alone and other diets. However, the maximum female-biased sex ratio was 76%, which was recorded for *P. plumifer* when fed on *C. vitis* only, while the minimum female-biased sex ratio was 53%, which was recorded for *E. scutalis* when fed on date palm pollen only.

## 3. Discussion

This study is the first documentation of the life history, predation capacity, fecundity, and life table parameters of *P. plumifer* and *E. scutalis* on the grape erineum mite as prey. It shows that the grape erineum mite is an acceptable prey and of high nutritional value for *P. plumifer* and *E. scutalis* resulting in a short developmental time and high fecundity rate. They were also able to develop and reproduce successfully when fed on fresh date palm pollen or on a mixed diet of grape erineum mite and date palm pollen. This is a significant step in the development of biocontrol strategies against the grape erineum mite. For both predators, larvae developed to the protonymphal stages without feeding. Non-feeding larvae behavior may be a mechanism for the avoidance of sibling cannibalism or reducing intraspecific competition. Similar findings have been observed in many phytoseiidae species [39].

The developmental time of the different life stages of *P. plumifer* on all three diets tested in the current study are considerably shorter than those stated by Moghadasi et al. 2006 for this predator when preying on spider mite, *Tetranychus urticae* Koch at 27 °C and 75–80% RH [40], or the eriophyid mite, *Rhyncaphytoptus ficifoliae* (Keifer) at 25 °C and 65% RH [25]. These researchers stated a mean total developmental time of *P. plumifer* females of 8.62 and 8.73 days on the respective prey species. The developmental time of female immatures of *P. plumifer* fed on fig spider mite *Eotetranychus hirsti* [41] at 35 °C and 60% RH are very close to the present results against *C. vitis*. On the other hand, the development of *P. plumifer* immature females was slightly longer in our study (6.37 days at 33 °C) than reported when fed on the eriophyid mite, *Aceria olivi* (5.67 d at 35 °C) [15]. In the present study, the developmental rate of *E. scutalis* was shorter (6.69 days) on a mixed date-palm-pollen–prey diet compared to date palm pollen alone, which is faster than on the other prey, including mites and insects such as *Oligonychus afrasiaticus*, *Eutetranychus orientalis*, *T. urticae*, *R. ficifoliae*, and *Insulaspis palidulla* (9.6, 8.19, 8.02, 7.00, and 6.75 days, respectively) [30,31,42], as well as pollens like sour orange pollen (*Citrus aurantium* L.), castor bean pollen (*Ricinus communis* L.), and alfalfa pollen (*Medicago sativa* L.) (7.90, 6.98, and 8.94 days, respectively) [43]. Muñoz-Cárdenas et al. (2014) noted a positive influence of a mixed diet of eggs from whiteflies and spider mites on the developmental time and fecundity of predatory mite *Balaustium leanderi* compared to either food alone [44]. A positive effect of a mixed diet on the development, predation capacity, reproduction, and life history parameters has been reported for other predacious mites as well [45].

The survival rate of immature stages was not significantly affected by diet, and it exceeded 90% for both predators on all three diets. The findings of Vervaet et al. (2022) support our results. They revealed that the survival rate of *Pronematusu biquitus* and *Homeopronematus anconai* during the immature stages exceeded 83% for both mites on three diets [45].

The pre-oviposition periods of *P. plumifer* and *E. scutalis* were close to those stated by Kasap and Şekeroğlu (2004) [28] and Hamedi et al. [46]. When *P. plumifer* and *E. scutalis* were fed on a mixed diet of *C. vitis* and date palm pollen, there was a significant increase in oviposition period, fecundity, and adult female longevity. Subsequently, predators’ performance was strong. The oviposition period and female longevity of *P. plumifer* were parallel to the findings stated by Kouhjani-Gorji et al. (2012) [47], Louni et al. (2014) [25], Shakarami and Bazgir (2017) [41], and Al-Azzazy and Alhewairini (2020b) [15] for *P. plumifer* feeding on *T. urticae*, *R. ficifoliae*, *E. hirsti*, and *Tegolophus hassani*.

The oviposition duration and adult female longevity of *E. scutalis* (22.49 and 28.96) on a mixed diet were close to that reported against *Panonychus citri* (21.3, 28.6) [28] and (20.22, 29.57) when *E. scutalis* fed on Crawlers of *Bemisia tabaci* [48]. Although the addition of date palm pollen to the rearing units lowered the grape erineum mite predation by both predators, it substantially increased the fecundity of the predatory mites, and oviposition was always higher. The highest oviposition was obtained when *P. plumifer* was fed on a mixed diet (56.81 eggs/female). This value was higher when compared to mites that fed on *T. urticae* (49.10 eggs/female) [49], on *R. ficifoliae* (28.47 eggs/female) [25], on *E.hirsti* (35.71 eggs/female) [41], and on *Oxycenus niloticus* (50.80 eggs/female) [15]; furthermore, the results of Al-Azzazy and Alhewairini (2020b) for *P. plumifer* fed on *A. olivi* (57.46 eggs/female) [15] were higher than that obtained in this study. The maximum oviposition of *E. scutalis* was (44.16 eggs/female), which was higher than reported by Kasap and Şekeroğlu (2004) [28] (39.7 eggs/female) with feeding on *P. citri*. Also, *E. scutalis* has shown total fecundity of (17.13, 19.96, 23.16, 25.92, and 26.52 eggs/female) on golden shower tree pollen, caper bush pollen, *Tetranychus turkestani*, date palm pollen, and cattail pollen, respectively [35]. Moreover, the results of Bounfour and McMurtry (1987) for this predator fed on *Tetranycus pacificus* eggs (59.0 eggs/female at 35 °C) [27] were considerably higher than the value estimated in the current study. The high fecundity for both predators in the current study might be due to feeding on mixing prey with pollen. In *Amblydromalus limonicus*, Samaras et al. (2021) showed that mixing prey with pollen resulted in higher fecundity and *r_m_* values, thus enhancing the medium-to long-term thrips-control potential [49].

In the current study, the addition of date palm pollen significantly lowered the predation rate of grape erineum mites by *P. plumifer* and *E. scutalis*, 59.81 and 52.77%, respectively. These results agree with those of Vervaet et al. (2022), who stated that *H. anconai* devoured fewer *Aculops lycopersici* adults in the presence of *Typha latifolia* L. pollen [45]. Similar findings have been obtained by Samaras et al. (2021), who showed the different effects of *Zea mays*, *Typha angustifolia* and *Pinus brutia* pollen on the predation rate of *A. limonicus* [49]. In the absence of date palm pollen, *P. plumifer* immature females consumed 106.86 prey of *C. vitis* in this study, while they devoured 108 individuals of *Aceria ficus* [50], 58.29 individuals of *R. ficifoliae* [25], and 127.46 individuals of *T. hassani*, 135.83 of *O. niloticus*, and 143.82 of *A. olivi* [15]. *Euseius scutalis* immature females devoured 96.62 prey of *C. vitis* in this study, while they consumed 40.78 individuals of *R. ficifoliae* and 65.30 of *A. ficus* [25].

The life table parameters of both predatory mites were clearly affected by diet. Several biological studies have confirmed that high-quality food sources result in higher values in life table parameters [51,52]. The rates of population growth were promising for *P. plumifer* fed on mixed stages of *C. vitis* and date palm pollen. This was proven by (*r_m_*), which was 0.251. The reported intrinsic rate of increase for *P. plumifer* on *T. urticae* (0.200 at 26 °C) [31], *E. hirsti* (0.180 at 30 °C), *R. ficifoliae* (0.154 at 25 °C), and corn pollen (0.112 at 27 °C) [24] was lower than that obtained in this study when *P. plumifer* fed on mixed stages of *C. vitis* and date palm pollen. Kouhjani-Gorji et al. (2012) estimated (*r_m_*) of 0.244 for *P. plumifer* fed on *T. urticae* at 35 °C [47]. Also Al-Azzazy and Alhewairini estimated (*r_m_*) of 0.277, 0.288 and 0.298 for *P. plumifer* fed on *T. hassani*, *O. niloticus* and *A. olivi* at 35 °C, respectively [15]. This is somewhat higher than our estimate. The values of (*R_o_*), (*r_m_*), and (λ) of *P. plumifer* at 33 °C and 60% RH in the current study are higher than reported for *N. barkeri* against *C. vitis* at 35 °C and 50% RH [2]. *P. plumifer* performed better on *C. vitis* than *N. barkeri*, and this could be due to the moderate humidity level used in this study. In view of this, *P. plumifer* could be a useful biocontrol agent for *C. vitis*.

In the case of *E. scutalis* against *T. urticae*, *E. orientalis*, and *Oligonychus afrasiaticus* at 26 °C, the life table parameters (*r_m_*, λ, *R_o_*) values were 0.220, 0.175, and 0.161; 1.247, 1.192, and 1.175; and 26.73, 13.24, and 13.60, respectively [31]. On alfalfa pollen (*Medicago sativa* L.), 0.153, 1.150, and 18.51 [43] and on eriophyid mite, *A. ficus* and *R. ficifoliae*, at 28 °C, 0.218, 1.243, and 12.51 and 0.215, 1.240, and 12.02, respectively [30], were seen, while in the current study, they were 0.229, 1.254, and 26.82 at 33 °C. This indicates that *E. scutalis* performs well on *C. vitis* as a generalist predator. The rather high intrinsic rate of the natural increase (*r_m_*) of *P. plumifer* and *E. scutalis* reached with a mixed diet could be highly favorable for mass production and augmentative release purposes. Therefore, date palm pollen can be considered an optimal supplementary food for *P. plumifer* and *E. scutalis*. In addition, any short-term negative impacts on predation rate due to preference of the pollen and floral nectar over the prey or predator satiation have been shown to be eventually overbalanced by an increase in the predation rate at the population level, i.e., long-term positive impacts of the mixed diet.

## 4. Materials and Methods

### 4.1. Stock Culture of Predators

The individuals of *Phytoseius plumifer* and *E. scutalis* were collected from unsprayed (for the previous 3 years) vineyards of Buraidah city (26.300366° N, 43.789661° E), Saudi Arabia, in the summer of 2021. The grape leaves containing the predators were cut and transferred to the laboratory. All predators were maintained separately on rearing units made of common bean (*Phaseolus vulgaris* L.) leaves which were placed underside facing up on daily moistened cotton in plastic trays (6 × 12), in an incubator at 33 ± 1 °C, 60% ± 5% RH, and with a 16:8 (L:D) h photoperiod. The edges were bordered with water-saturated tissue paper to provide the predators with water and prevent them from escaping. The grape leaves infested with *C. vitis* were used to feed the predatory mites five times a week, adding date palm pollen with a thin paintbrush as a supplementary food. Water was added to the cotton every day to keep the arena humid. Predator eggs were collected and transferred individually to the new rearing arenas to obtain cohorts of individuals of the same age.

Fifteen microscope slides were prepared with each species to confirm their identification. The identification of both the predators was confirmed according to Chant and McMurtry (2007) [53].

### 4.2. Stock Cultures of Prey

Grape erineum mites were collected from a vineyard grown at the Experimental Research Station of the College of Agriculture and Food (Qassim, Saudi Arabia). The specimens of *C. vitis* were transferred to the laboratory and reared on grape seedlings as a permanent source of prey, kept in a climate room at 30 ± 1 °C, 60% ± 5% RH, and with a 16:8 (L:D) h photoperiod. All the usual agriculture practices such as fertilization and irrigation were followed.

### 4.3. Experimental Set-Up

All experiments were performed in an incubator at 33 ± 1 °C, with a 12:12 h (L:D) photoperiod, at an average daily relative air humidity of 60 ± 5%. Freshly excised grape leaf disks (3 × 3 cm) were used as rearing arenas. The leaves were placed with the lower surface facing up on daily moistened cotton inside a Pyrex^®^ Petri dish (5 cm in diameter, 2 cm high). The edges were bordered with water-saturated cotton to provide water and avoid mite escape.

### 4.4. Pollen Collection

Fresh date palm pollen (*Phoenix dactylifera* L.) was collected from trees planted in the Qassim region, Saudi Arabia, and oven-dried at 25 °C for one day and then stored at −18 °C. Before use in the trials, a small amount of date palm pollen was refrigerated at 4 °C for up to 2 weeks for early use in the experiments.

### 4.5. Effects of Diet on Life History Parameters of P. plumifer and E. scutalis

For each predator, 30 to 40 fresh eggs (less than 7 h old) from the stock culture were transferred individually to the grape leaf arena. The immature developmental time, survival, sex ratio, fecundity, and longevity of *P. plumifer* and *E. scutalis* were determined by feeding them with one of the following food sources: (1) date palm pollen, (2) mixed stages of *C. vitis*, and (3) mixed stages of *C. vitis* plus date palm pollen. The mixed stages of *C. vitis* were provided by a carefully examined small disk (0.5 cm in diameter) of severely infested grape leaves to record the total number of mites per disk before introducing it into the rearing arenas on the grape leaf disk for 24 h, after which the number of consumed preys was recorded. Date palm pollen grains were placed on the rearing arenas using a fine brush, three times per week. Observations were made twice a day to determine the developmental time (egg, larva, protonymph, and deutonymph) and the survival of immature stages. For each rearing arena, after the emergence of adults, a single male was put in the new female’s rearing arena for mating. Males were then transferred into new leaf disks and individually reared until the end of their lifespan. Some cotton fibers were stuck on grape leaf disks to provide a suitable place for oviposition. The experimental units were monitored twice a day for any changes recorded until the death of the last female. This monitoring allowed us to determine the pre-oviposition, oviposition, and post-oviposition periods and the female and male longevity and fecundity. Once the adult stage was reached, the sex of the predators was determined. Whenever the quality of the rearing arena began to deteriorate, it was replaced with a fresh leaf disk. To test the sex ratio, daily, the eggs laid were placed individually into separate rearing units with a fine brush, and the hatched larvae were reared to adulthood to determine the sex ratio of the progeny.

### 4.6. Statistical Analysis

To assess immature development, pre-oviposition and post-oviposition periods, fecundity, adult longevity, predation, and life span of *P. plumifer* and *E. scutalis* and the effect of three food sources on these parameters, data were compared with analysis of variance (ANOVA), using SAS computer program version 9.2 (SAS, 2008). Means were separated by Duncan’s multiple range test (DMRT) at *p* < 0.05. The means of survival percentages were separated using Tukey’s honestly significant difference test (Tukey’s HSD test). The life table parameters for both predators were constructed based on Birch (1948). The sex ratio for both predators was analyzed using a Chi-square test.

## 5. Conclusions

In conclusion, our results demonstrate that the grape erineum mite *C. vitis* is a suitable prey for both phytoseiids *P. plumifer and E. scutalis,* making them promising biocontrol agents of that pest. Furthermore, the addition of date palm pollen to the diet of *C. vitis* substantially increased the reproductive performance of both predators. Therefore, it can be concluded that the mixed diet of *C. vitis* and date palm pollen are good candidates for the mass rearing of *P. plumifer* and *E. scutalis* for use in augmentative biocontrol programs. Moreover, the addition of date palm pollen as an optimal supplementary food source can be an effective tool to boost the populations of *P. plumifer* and *E. scutalis* and enhance biocontrol even in the presence of a low grape erineum mite population.

## Figures and Tables

**Table 1:** Average development duration in days of the immature stages of *Phytoseius plumifer* and *Euseius scutalis* feeding on three diets at 33 ± 1 °C, 60% ± 5% RH.

Predator Species	Diet	Sex	Egg	Larva	Protonymph	Deutonymph	Overall Developmental Time
*Phytoseius plumifer*	Date palm pollen.	Female	2.31 ± 0.22 a	1.05 ± 0.18 a	1.39 ± 0.14 a	1.62 ± 0.16 a	6.37 ± 0.65 a
		Male	2.25 ± 0.18 a	1.17 ± 0.12 a	1.31 ± 0.12 a	1.52 ± 0.14 a	6.25 ± 0.60 a
	mixed stages of *C. vitis*	Female	2.38 ± 0.18 a	1.05 ± 0.20 a	1.30 ± 0.18 a	1.56 ± 0.14 a	6.29 ± 0.47 a
		Male	2.35 ± 0.16 a	1.02 ± 0.10 a	1.24 ± 0.11 a	1.53 ± 0.12 a	6.14 ± 0.52 a
	mixed stages of *C. vitis* + date palm pollen	Female	2.30 ± 0.14 a	1.10 ± 0.04 a	1.29 ± 0.05 a	1.47 ± 0.05 a	6.16 ± 0.45 a
		Male	2.30 ± 0.13 a	1.08 ± 0.05 a	1.25 ± 0.04 a	1.43 ± 0.04 a	6.06 ± 0.52 a
*Euseius scutalis*	Date palm pollen	Female	2.36 ± 0.25 a	1.25 ± 0.20 a	2.26 ± 0.28 a	2.57 ± 0.18 a	8.44 ± 0.72 a
		Male	2.32 ± 0.22 a	1.22 ± 0.18 a	2.23 ± 0.20 a	2.84 ± 0.16 a	8.25 ± 0.62 a
	mixed stages of *C. vitis*	Female	2.32 ± 0.15 a	1.23 ± 0.17 a	1.64 ± 0.18 b	1.91 ± 0.12 b	7.10 ± 0.52 b
		Male	2.30 ± 0.12 a	1.22 ± 0.15 a	1.62 ± 0.16 b	1.85 ± 0.08 b	6.99 ± 0.66 b
	mixed stages of *C. vitis* + date palm pollen	Female	2.30 ± 0.18 a	1.21 ± 0.09 a	1.51 ± 0.09 b	1.67 ± 0.05 b	6.69 ± 0.64 b
		Male	2.28 ± 0.14 a	1.23 ± 0.07 a	1.50 ± 0.11 b	1.64 ± 0.07 b	6.65 ± 0.71 b

Different letters in each column denote significant difference within each species (ANOVA followed by Duncan’s multiple range test: *p* < 0.05).

**Table 2:** Survival of immature stages of *Phytoseius plumifer* and *Euseius scutalis* feeding on three diets at 33 ± 1 °C, 60% ± 5% RH.

Predator Species	Diet	Stage Specific Survival (% ± SE)				Survival to Adulthood (% ± SE)
		Egg	Larva	Protonymph	Deutonymph	
*Phytoseiu splumifer*	Date palm pollen	93.25 ± 3.89	91.25 ± 4.12	91.08 ± 4.54	90.91 ± 4.19	90.14 ± 4.12
	*C. vitis*	94.54 ± 4.11	96.30 ± 3.96	95.16 ± 3.28	93.52 ± 3.85	92.23 ± 3.43
	*C. vitis* + pollen	97.35 ± 3.81	98.54 ± 3.35	97.10 ± 3.49	94.29 ± 3.18	94.12 ± 3.81
*Euseius scutalis*	Date palm pollen	92.24 ± 3.60	90.60 ± 4.87	89.42 ± 4.74	90.17 ± 3.61	90.37 ± 4.15
	*C. vitis*	93.97 ± 4.51	92.67 ± 4.45	92.25 ± 3.45	91.05 ± 3.53	91.80 ± 3.25
	*C. vitis* + Pollen	95.64 ± 3.78	94.30 ± 3.61	93.85 ± 3.56	92.14 ± 4.23	91.15 ± 2.89

No significative differences according to the Tukey HSD test.

**Table 3:** Average development duration in days of *Phytoseius plumifer* and *Euseius scutalis* adults feeding on three diets at 33 ± 1 °C, 60% ± 5% RH.

Predator Species	Diet	Pre Oviposition	Oviposition	Post Oviposition	Longevity		Life Span	
					Female	Male	Female	Male
*Phytoseius plumifer*	Date palm pollen	2.85 ± 0.18 a	21.16 ± 0.1.24 a	4.47 ± 0.24 a	28.48 ± 1.16 a	26.89 ± 1.25 a	34.85 ± 1.35 a	33.14 ± 1.88 a
	mixed stages of *C. vitis*	2.79 ± 0.19 a	25.96 ± 0.79 b	4.38 ± 0.30 a	33.13 ± 0.96 b	31.53 ± 1.82 b	39.42 ± 1.46 b	37.42 ± 1.42 b
	mixed stages of *C. vitis* + date palm pollen	2.21 ± 0.14 b	26.87 ± 0.92 b	4.35 ± 0.38 a	33.43 ± 1.04 b	32.27 ± 0.1.14 b	39.59 ± 1.36 b	38.43 ± 1.69 b
*Euseius scutalis*	Date palm pollen	4.37 ± 0.31 a	16.58 ± 0.95 a	3.16 ± 0.34 a	24.11 ± 0.98 a	19.47 ± 2.34 a	32.55 ± 1.31 a	27.72 ± 2.51 a
	mixed stages of *C. vitis*	3.30 ± 0.24 a	21.12 ± 1.06 b	3.88 ± 0.46 a	28.30 ± 1.14 b	25.95 ± 0.98 b	35.40 ± 1.44 b	32.94 ± 1.70 b
	mixed stages of *C. vitis* + date palm pollen	2.45 ± 0.16 c	22.49 ± 0.83 b	4.02 ± 0.20 a	28.96 ± 1.26 c	27.43 ± 0.1.05 c	35.65 ± 1.33 c	34.08 ± 1.51 c

Different letter denotes significant difference within species (ANOVA followed by Duncan’s multiple range test: *p* < 0.05).

**Table 4:** Fecundity of *Phytoseius plumifer* and *Euseius scutalis* feeding on three diets at 33 ± 1 °C, 60% ± 5% RH.

Diet	*Phytoseius plumifer*		*Euseius scutalis*	
	Total Fecundity ± SD	Daily Fecundity	Total Fecundity ± SD	Daily Fecundity
Date palm pollen	35.48 ± 1.12 Aa	1.67 ± 0.08	24.55 ± 1.07 Ab	1.48 ± 0.04
*C. vitis*	53.94 ± 1.28 Bb	2.07 ± 0.06	40.09 ± 1.22 Bc	1.89 ± 0.09
*C. vitis* + Pollen	56.81 ± 1.30 Bd	2.11 ± 0.09	44.16 ± 1.35 Be	1.96 ± 0.05

The capital letter denotes the significance within the same column, and the small letter denotes the significance within the same row at *p* < 0.05.

**Table 5:** Predation rate by different stages of *Phytoseius plumifer* and *Euseius scutalis* feeding on grape erineum mite, *Colomerus vitis* at 33 ± 1 °C, 60% ± 5% RH.

Predatory Stage	Sex	*P. plumifer*		*E. scutalis*	
		No. of Attacked Mite Individuals			
		Total Average Mean ± SD	Daily Rate, Mean ± SD	Total Average, Mean ± SD	Daily Rate, Mean ± SD
Protonymph	Female	43.84 ± 1.68	33.72 ± 1.52	37.34 ± 2.43	22.76 ± 1.64
	Male	38.55 ± 2.12	31.08 ± 2.04	36.27 ± 2.50	22.38 ± 1.20
Deutonymph	Female	63.02 ± 2.45	40.39 ± 2.30	59.28 ± 3.16	31.03 ± 2.36
	Male	58.00 ± 1.89	37.90 ± 2.11	58.14 ± 2.70	31.42 ± 1.85
Pre-oviposition	Female	241.48 ± 3.15	86.55 ± 2.07	239.44 ± 3.65	72.55 ± 2.25
Oviposition	Female	2931.40 ± 16.62 a	112.91 ± 4.53 a	1994.52 ± 12.40 b	94.43 ± 4.16 b
Post-oviposition	Female	245.89 ± 2.30	56.13 ± 2.26	165.52 ± 3.35	42.66 ± 2.51
Longevity	Female	3418.77 ± 14.50 a	103.19 ± 3.08 a	2355.87 ± 15.85 b	83.24 ± 3.49 b
	Male	2845.36 ± 12.53	90.24 ± 3.60	1882.93 ± 14.14	72.56 ± 4.14
Life span	Female	3525.63 ± 16.47 a	89.43 ± 3.14 a	2452.49 ± 10.53 b	69.27 ± 3.30 b
	Male	2941.91 ± 12.90	78.61 ± 2.82	1977.34 ± 13.57	60.02 ± 3.14

Means followed by different letters in each row for total average and daily rate separately denote significant differences (ANOVA followed by Duncan’s multiple range test: *p* < 0.05) (The comparation is made only with females).

**Table 6:** Predation rate by different stages of *Phytoseius plumifer* and *Euseius scutalis* feeding on a mixed diet of the grape erineum mite, *C. vitis* plus date palm pollen at 33 ± 1 °C, 60% ± 5% RH.

Predatory Stage	Sex	*P. plumifer*		*E. scutalis*	
		No. of Attacked Mite Individuals			
		Total Average, Mean ± SD	Daily Rate, Mean ± SD	Total Average, Mean ± SD	Daily Rate, Mean ± SD
Protonymph	Female	22.86 ± 1.21	17.72 ± 0.96	19.85 ± 0.71	13.14 ± 0.64
	Male	22.47 ± 1.06	17.97 ± 0.80	18.61 ± 0.75	12.40 ± 0.87
Deutonymph	Female	33.88 ± 1.12	23.04 ± 1.91	26.75 ± 1.18	16.01 ± 1.05
	Male	32.72 ± 1.36	22.88 ± 1.25	20.81 ± 0.98	12.68 ± 0.90
Pre-oviposition	Female	143.07 ± 2.11	64.73 ± 1.16	129.55 ± 1.23	52.87 ± 1.08
Oviposition	Female	1769.43 ± 10.42 a	65.85 ± 1.41 a	1037.83 ± 3.52 b	46.14 ± 3.16 b
Post-oviposition	Female	139.50 ± 2.09	32.06 ± 0.96	80.27 ± 1.46	19.96 ± 0.84
Longevity	Female	2052.00 ± 11.34 a	61.38 ± 2.59 a	1247.65 ± 16.25 b	43.08 ± 2.14 b
	Male	1439.65 ± 13.68	44.61 ± 1.83	1003.23 ± 10.72 b	36.56 ± 2.30 b
Life span	Female	2108.74 ± 14.23 a	53.26 ± 1.17 a	1294.25 ± 14.95	36.30 ± 2.48 b
	Male	1494.84 ± 10.89	38.89 ± 1.34	1042.65 ± 7.84 b	30.59 ± 2.27

Means followed by different letters in each row for total average and daily rate separately denote significant differences (ANOVA followed by Duncan’s multiple range test: *p* < 0.05). (The comparation is made only with females).

**Table 7:** Population growth parameters of *Phytoseius plumifer* and *Euseius scutalis* feeding on three diets at 33 ± 1 °C, 60% ± 5% RH.

Parameters	*C. vitis*		*C. vitis* + Pollen		Date Palm Pollen	
	*P. plumifer*	*E. scutalis*	*P. plumifer*	*E. scutalis*	*P. plumifer*	*E. scutalis*
Net reproduction rate (*R_o_*)	27.65	24.31	29.12	26.82	20.46	19.10
Mean generation time (*T*) (days)	18.57	19.65	17.21	18.72	22.36	25.48
Intrinsic rate of increase (*r_m_*)	0.242	0.211	0.251	0.229	0.194	0.175
Finite rate of increase (λ)	1.246	1.212	1.377	1.285	1.194	1.186
Sex ratio	0.76	0.73	0.73	0.70	0.60	0.53
	(f = 23; m = 7)	(f = 22; m = 8)	(f = 22; m = 8)	(f = 21; m = 9)	(f = 18; m = 12)	(f = 16; m = 14)

## Data Availability

Data are contained within the article.
